# Medical School Ranking and Neighborhood Characteristics of Initial Practice Location Among Physicians

**DOI:** 10.1001/jamanetworkopen.2025.12474

**Published:** 2025-05-28

**Authors:** Navapat Nananukul, Mayank Kejriwal

**Affiliations:** 1 Information Sciences Institute, University of Southern California, Los Angeles

## Abstract

This cross-sectional study examines the association of physicians’ gender, medical school, and specialty with their likelihood of initially practicing in socioeconomically deprived areas.

## Introduction

The US has experienced a chronic shortage of physicians in certain geographic regions, especially those that may be deprived.^1^ Understanding the factors influencing the practice locations of newly licensed physicians is important for addressing health inequities.^2^ Although previous studies have examined overall physician distribution, less is known about the associations between physicians’ characteristics and the likelihood of initially practicing in socioeconomically deprived areas, particularly for physicians from top-tier medical schools, who often have access to more resources and advanced training and whose placement in underserved areas may be valuable for closing health equity gaps.^3,4^ This study quantifies the association between physicians’ characteristics, including stated gender, medical school, and specialty, with their likelihood of practicing in socioeconomically deprived areas.

## Methods

Physician data from the Centers for Medicare & Medicaid Services National Downloadable File was combined with socioeconomic data from the Area Deprivation Index (ADI) Neighborhood Atlas (eMethods and eTable in Supplement 1). ADI is a validated measure of neighborhood socioeconomic disadvantage, incorporating factors such as income, education, employment, and housing quality.^5^ We performed a cross-sectional study by analyzing 2 cohorts of newly licensed physicians (2015 and 2020) for which ADI ranks are available. This study analyzed publicly available, open-government data and was therefore not considered human participants research. Institutional review board approval and informed consent were not required in accordance with federal guidelines (45 CFR §46). STROBE reporting guidelines were followed.

We used regression analysis to measure associations between physicians’ characteristics and likelihood of practicing in high ADI-ranked (ie, socioeconomically deprived) areas. The primary outcome variable was binary, indicating whether a physician practiced in an area at or above the 80th percentile of the national ADI ranking, vs lower ADI area. Factors include gender, specialty group, and graduation from top-ranked medical schools (eMethods in Supplement 1).

## Results

Of the 83 833 physicians in 2015, those from top-ranked medical institutions had 52% lower odds of practicing in socioeconomically deprived areas compared with physicians from lower-ranked institutions (odds ratio [OR], 0.48; 95% CI, 0.43-0.54; *P* < .001), with similar findings in 2020 (OR, 0.50; 95% CI, 0.41-0.61; *P* < .001) (Table). Compared with primary care physicians, specialists from 11 of 13 specialties had lower odds of practicing in socioeconomically deprived areas in 2015, whereas 8 of 13 had lower odds in 2020.

**Table.** Association of Sociodemographic and Physician Specialties With Practicing in Socioeconomically Deprived Area

Variables	OR (95% CI)		AME (95% CI)	
	2015	2020	2015	2020
Sociodemographic characteristics				
Male sex (reference, female)	1.08 (1.04 to 1.13)	0.98 (0.93 to 1.02)	0.012 (0.006 to 0.018)	−0.003 (−0.010 to 0.003)
Top 20 medical schools, yes (reference, no)	0.48 (0.43 to 0.54)	0.50 (0.41 to 0.61)	0.498 (0.408 to 0.608)	−0.103 (−0.132 to −0.074)
Clinical specialties (reference, primary care)				
Advanced practice practitioners	1.24 (1.17 to 1.31)	1.11 (1.04 to 1.19)	0.033 (0.024 to 0.041)	0.016 (0.006 to 0.026)
Emergency medicine	1.21 (1.09 to 1.35)	1.74 (1.56 to 1.94)	0.029 (0.012 to 0.045)	0.082 (0.066 to 0.098)
Obstetrics or gynecology	0.88 (0.77 to 1.00)	1.66 (1.27 to 2.16)	−0.020 (−0.040 to 0.000)	0.075 (0.035 to 0.114)
Anesthesiology	0.72 (0.64 to 0.82)	0.72 (0.54 to 0.95)	−0.049 (−0.068 to −0.030)	−0.049 (−0.091 to −0.007)
Medical specialties	0.67 (0.62 to 0.72)	0.62 (0.51 to 0.74)	−0.061 (−0.073 to −0.050)	−0.072 (−0.098 to −0.045)
Ophthalmology or otorhinolaryngology	0.79 (0.68 to 0.92)	0.66 (0.51 to 0.86)	−0.036 (−0.058 to −0.013)	−0.062 (−0.100 to −0.023)
Other specialties (alternative)	0.99 (0.89 to 1.09)	0.87 (0.77 to 0.98)	−0.002 (−0.017 to 0.013)	−0.021 (−0.038 to −0.003)
Radiology	0.69 (0.62 to 0.77)	0.45 (0.41 to 0.50)	−0.056 (−0.071 to −0.040)	−0.124 (−0.196 to −0.052)
Rehabilitation or therapy	0.55 (0.50 to 0.61)	0.55 (0.50 to 0.61)	−0.090 (−0.105 to −0.074)	−0.118 (−0.133 to −0.102)
Pathology	0.74 (0.57 to 0.96)	1.82 (0.52 to 6.39)	−0.046 (−0.085 to −0.007)	0.089 (−0.096 to 0.274)
Dentistry	0.87 (0.38 to 1.98)	0.45 (0.14 to 1.47)	−0.021 (−0.146 to 0.103)	−0.119 (−0.296 to 0.057)
Psychiatry	0.95 (0.87 to 1.05)	0.91 (0.80 to 1.04)	−0.007 (−0.021 to 0.007)	−0.014 (−0.033 to 0.005)
Surgery	0.89 (0.81 to 0.97)	1.13 (0.72 to 1.77)	−0.018 (−0.032 to −0.004)	0.018 (−0.049 to 0.085)

Abbreviations: AME, average marginal effect; OR, odds ratio.

The distribution of physicians across the US was different when comparing physicians from top-ranked institutions and lower-ranked institutions. The Figure shows that the number of physicians from top-ranked institutions was negatively associated with the location’s socioeconomic deprivation, even after controlling for population. In contrast, physicians from lower-ranked institutions exhibited the inverse trend.

**Figure.**
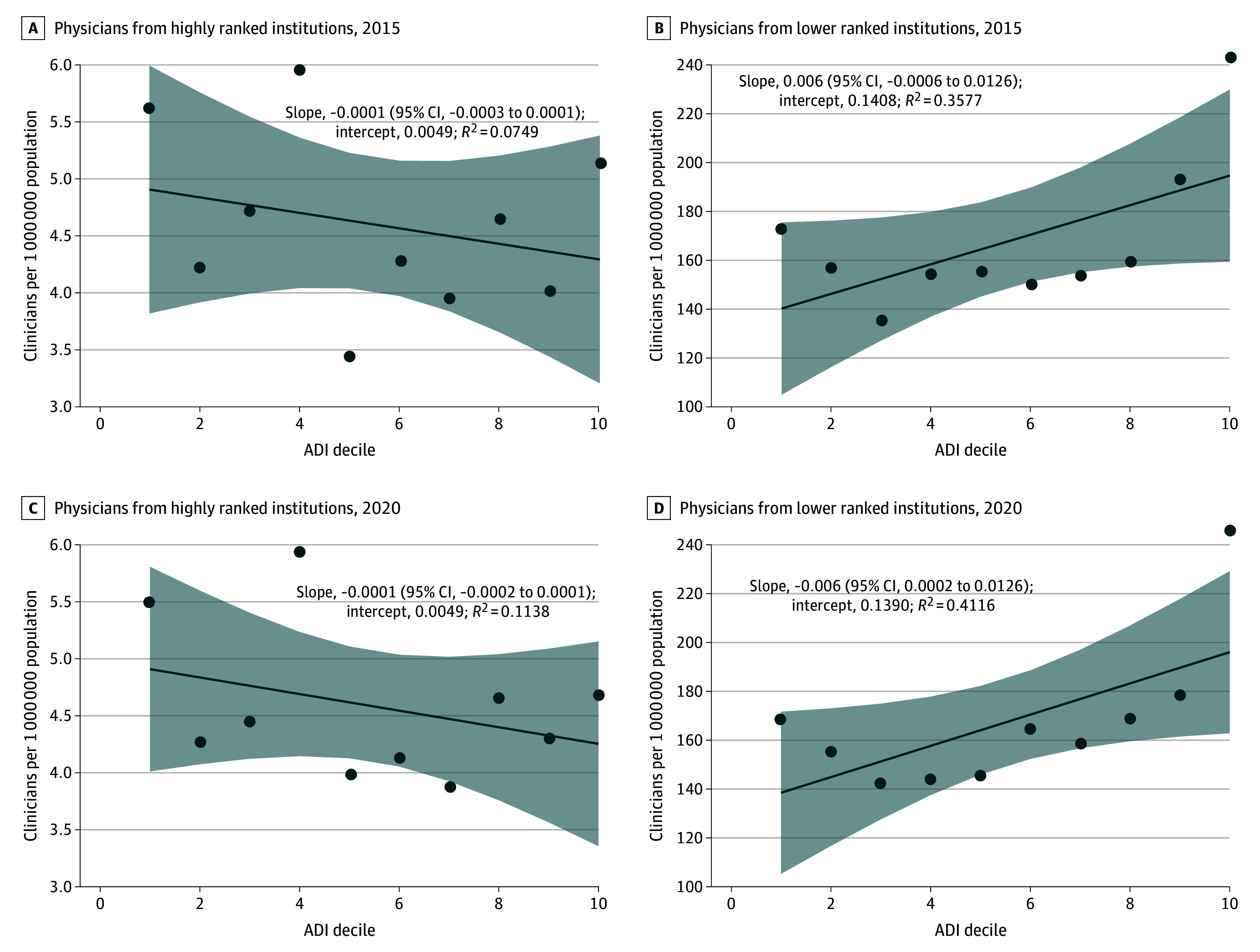
Association of Physician Density With Area Deprivation Index (ADI) Graphs illustrate the distribution of physicians per 1 000 000 population from low to high ADI deciles (socioeconomically deprived areas). The far-right area in each plot above the eighth decile indicates the ADI range that was defined in the main analysis as representing socioeconomically deprived areas (≥80th percentile). A positive slope suggests a higher physician density in areas with a higher ADI decile, whereas a negative slope indicates a lower density in these areas. Each scatter plot includes a trend line with shaded 95% CIs, slope estimates, intercepts, and *R*^2^ values.

## Discussion

This cross-sectional study found that an area’s ADI was significantly associated with newly licensed health care physicians’ decision to practice in that area, and the association was especially acute for physicians from top-ranked institutions and certain specialties. One possible reason is that factors associated with high ADI areas—limited resources, lower income potential, or fewer professional development opportunities—may demotivate ambitious physicians from working in these areas.^6^ However, the analysis does not permit a causal investigation. Regardless of the reason, the data show that many medical specialties have lower representation in socioeconomically deprived areas compared with primary care, which may contribute to disparities in specialized health care access in deprived areas. The findings underscore the need for targeted initiatives to incentivize physicians, particularly specialists and top medical graduates, to practice in socioeconomically disadvantaged regions.

This study has limitations. Although medical school ranking was considered, data on the actual residency training location and race and ethnicity were unavailable. These 2 factors potentially affect initial practice preferences. Physicians’ childhood environments and financial debt could also impact their career location decisions. Finally, the study year of 2020 overlapped with the COVID-19 pandemic, which disproportionately affected underserved communities and may have introduced bias.

## Notes

Supplement 1.
eMethods.
**eTable.** Percentage of records before and after removing missing ADI records
eReferences

Supplement 2.
Data Sharing Statement
