# Supplementary material for: Medical School Ranking and Neighborhood Characteristics of Initial Practice Location Among Physicians
Source: JAMA Netw Open. 2025 May 28;8(5):e2512474. doi: 10.1001/jamanetworkopen.2025.12474 (PMC12120638; doi:10.1001/jamanetworkopen.2025.12474)
Supplement: Supplement 1. — eMethods. eTable. Percentage of records before and after removing missing ADI records eReferences [file jamanetwopen-e2512474-s001.pdf]

Supplemental Online Content

Nananukul N, Kejriwal M. Medical school ranking and neighborhood characteristics of initial practice location among physicians. *JAMA Netw Open*. 2025;8(5):e2512474.
doi:10.1001/jamanetworkopen.2025.12474

eMethods
eTable. Percentage of records before and after removing missing ADI records
eReferences

This supplemental material has been provided by the authors to give readers additional information about their work.

© 2025 Nananukul N et al. *JAMA Network Open*.

eMethods.

The data sources, code for analyses and visualizations are documented in the corresponding Jupyter notebooks on the project's GitHub page https://github.com/navapatn/JAMA-affluence-placement. This document summarizes all steps required for conducting the analysis.

Data sources

a. The Doctors and Clinicians national downloadable file

Clinician or Physician data was downloaded from *The Centers for Medicare & Medicaid Services* [1] on August 20, 2024. This dataset provides individual Physician-level data where each record is unique based on a combination of the following identifiers: NPI, Individual Enrollment ID, Organization PAC ID, and Address ID. For each unique Physician, we selected the earliest record in the dataset based on graduation year (grad_year) to obtain their first practice placement.

b. Area Deprivation Index (ADI) Data

We downloaded the The Area Deprivation Index (ADI) [2,3] on September 10, 2024. The data is provided by the Center for Health Disparities Research, University of Wisconsin. We used two versions available on the website: 2015 and 2020. We utilized the census block group level composite ADI index, which reflects 17 factors from the American Community Survey on income, education, employment, and housing, calculated via principal components methodology. An interactive overview of ADI data is provided in the official website [4].

© 2025 Nananukul N et al. *JAMA Network Open*.

C. The U.S. Census bureau Data

We used the interactive interface provided by the U.S. Census Bureau data [5] to obtain population estimates at the ZIP code level. We manually filtered the data from the interactive interface and converted it into a .csv format readable by Python's CSV package.

Data preprocessing

Physician data: We selected specific fields from the physicians data for analysis. The main variables chosen were gender (*gndr*), medical school (*Med_sch*), primary specialization (*pri_spec*), state (*states*), and ZIP code *(zip_code*). To ensure that we only included newly licensed physicians, we selected the first entry in the dataset for each National Provider Identifier (NPI) ID, representing physicians at the start of their practice and reducing selection bias. We combined the physicians data with population estimates at the ZIP code level from U.S. Census Bureau data by joining on the 5-digit ZIP code. Additionally, we utilized a list of the top 20 institutions to define highly ranked medical schools. This list was obtained from the US News medical school ranking [6]. We flagged each physician as either: 1: the physicians is from a highly-ranked medical school or 0: the physician is not from a highly-ranked medical school. To reduce the chance of misclassification, we manually edited and matched the institution names from the external list with those in the Medicare dataset. Furthermore, the variable Primary Specialization originally had 91 unique values, which we grouped into 13 categories. We prompted GPT-4o to assist in grouping the medical specializations. The prompt example and dictionary output by GPT-4o are provided in the Jupyter notebook

© 2025 Nananukul N et al. *JAMA Network Open*.

02-data-preprocessing.ipynb. In total, we include 83,833 physicians from the 2015 and 70,290 from the 2020 cohort.

ADI data: To combine the ADI data with the physician data, we used the USPS Crosswalk API [7] as our main mapper between census tracts and ZIP codes. We called the USPS Crosswalk API by selecting a parameter that provides a mapping from census tract to ZIP code (type 6 as documented in the USPS Crosswalk API documentation), along with other parameters such as the year and the 11-digit census tract code from the ADI data. This mapping allowed us to combine the ADI data with the physician data at the ZIP code level. Furthermore, we defined socioeconomically deprived areas by using the 80th percentile of the ADI as a threshold. Any area with an ADI rank higher than this threshold was flagged as a socioeconomically deprived area. This cutoff was selected based on our literature review. For example, in [8], it was noted that researchers commonly divided the ADI using percentile thresholds (e.g., tertiles, quartiles, or quintiles) to distinguish highly deprived areas. Second, earlier studies published in JAMA [9,10], defined highly disadvantaged areas using an 80th percentile cutoff (or equivalently, by selecting the last quintile). Based on these considerations, we chose the 80th percentile as our main threshold.

Missing data: When combining ADI data with Medicare data, some ZIP codes did not have a perfect match between the two datasets. In these cases, we removed them from the analysis since an accurate ADI could not be determined. Out of a total of 126,761 ZIP codes, we removed approximately 8,000 (6%) from the dataset. To assess the impact of excluding records with missing ADI data, we compared key descriptive statistics between records with complete ADI data and those with missing ADI values.

© 2025 Nananukul N et al. *JAMA Network Open*.

For example, the gender distribution was similar across groups (complete records: approximately 52% female, 48% male; missing records: approximately 51% female, 49% male). Likewise, the distribution of the top five states was comparable between the two groups. These comparisons indicate that the removal of approximately 6% of ZIP codes due to missing ADI data did not alter the overall distribution of key attributes in our sample.

To assess the impact of excluding records with missing ADI data, we compared key descriptive statistics between records with complete ADI data and those with missing ADI values. Variables such as genders and states were evaluated by calculating the distribution of the data before and after removing the missing ADI records. The **eTable** shows the percentage of records' values before and after removing missing data for genders and top five states**.** We confirmed that the exclusion of these records did not meaningfully alter the overall distribution of key attributes in our sample.

eTable: Percentage of records before and after removing missing ADI records.

**Variables (values)**	**All Records (%)**	**Record with Complete ADI (%)**
Gender (F)	35.42	35.47
Gender (M)	64.58	64.53
State (TX)	6.91	7.02
State (CA)	6.87	6.97
State(NY)	5.88	5.96
State(PA)	5.19	5.25
State(MI)	4.26	4.32

© 2025 Nananukul N et al. *JAMA Network Open*.

Sample size calculation for designing clinical research: To ensure that our study had sufficient power, we determined the minimum sample size required to compare an odds ratio from logistic regression to 1. The actual calculation was conducted using a standard online tool [11]. Specifically, we used the odds ratios for the variable *Top 20 medical schools* (reference: no) for both 2015 and 2020, as this variable is our primary finding. The calculator incorporates the proportion of samples from both groups, setting $\alpha = 0.05$ and $\beta = 0.20$, to determine the required sample size. The calculator computes the minimum detectable odds ratio per 1 SD increase in the continuous explanatory variable (with values closer to 1 indicating that the study is powered to detect smaller effect sizes). Using this approach, the required sample sizes were calculated as 102 for the 2015 cohort and 103 for the 2020 cohort. Hence, the actual study sample exceeds these numbers by a large margin (over 80,000 physicians), making a Type II error much less probable.

Data analysis

All analyses were performed using Python packages: pandas, statsmodels, lifelines, and matplotlib

Logistic Regression: We conducted logistic regression analyses to examine the association between physician characteristics and their likelihood of practicing in areas of high socioeconomic deprivation. The dependent variable was a binary indicator of whether a physician practiced in a socioeconomically deprived (high ADI) area (coded as 1) or not (coded as 0). The independent variables included: Gender (Male, with Female as the reference), 13 primary specialization groups (with Primary Care as the reference), Top 20 Institution (indicator coded as 1 if the physician graduated from a top

© 2025 Nananukul N et al. *JAMA Network Open*.

20 medical school and 0 otherwise), and Log of Total Population (the natural logarithm of the total population at the ZIP code level). The logistic regression models used Maximum Likelihood Estimation (MLE) to estimate the odds of physicians practicing in high ADI areas, both unadjusted and adjusted for potential confounders. Adjusted models controlled for gender, specialty, medical school rank, and population size based on prior research linking these factors to practice location choices. We reported odds ratios with 95% confidence intervals to indicate the precision of estimates, providing a clearer understanding of the independent association of each predictor with the outcome. This analysis was performed for both the 2015 and 2020 cohorts. Additionally, we conducted state-level and specialization-level regressions to assess whether physicians from top-20 medical schools had significantly lower odds of practicing in high ADI areas within each state and specialization.

Linear Regression: We performed additional linear regression analyses to backup our main findings by exploring the relationship between the Area Deprivation Index (ADI) deciles and number of physicians. Physician density was defined as the number of physicians per 1,000,000 population within each ADI decile for the years 2015 and 2020. Focusing on physicians from non-top 20 medical institutions, we conducted the least squares regression of physician density on ADI decile for each year. The regression models estimated the association between socioeconomic deprivation and physician density (per 1,000,000 population), providing slope, intercept, and R-squared values.

© 2025 Nananukul N et al. *JAMA Network Open*.

Data Visualization

We used the geopandas [12], matplotlib [13], and seaborn [14] packages for the creation of the scatter plots (Figure 1) .

Regression Result (Figure 1): We visualized the relationships that resulted from our linear regression analyses using scatter plots with fitted regression lines and 95% confidence intervals. The y-axis shows the number of physicians per 1,000,000 population and the x-axis shows the ADI deciles.

eReferences

1. https://data.cms.gov/provider-data/dataset/mj5m-pzi6#overview
2. Kind AJH, Buckingham W. Making Neighborhood Disadvantage Metrics Accessible: The Neighborhood Atlas. New England Journal of Medicine, 2018. 378: 2456-2458. DOI: 10.1056/NEJMp1802313. PMCID: PMC6051533.
3. University of Wisconsin School of Medicine and Public Health. 2024. Area Deprivation Index 2015 and 2020. Downloaded from https://www.neighborhoodatlas.medicine.wisc.edu/ September 10, 2024
4. https://www.neighborhoodatlas.medicine.wisc.edu/mapping
5. https://data.census.gov/table
6. https://www.usnews.com/best-graduate-schools/top-medical-schools
7. https://www.huduser.gov/portal/dataset/uspszip-api.html
8. Balio, Casey P., et al. "Use of the area deprivation index and rural applications in the peer-reviewed literature." *Rural Health Equity Research Center* (2024).
9. Hsia RY, Sarkar N, Shen Y. Provision of Stroke Care Services by Community Disadvantage Status in the US, 2009-2022. *JAMA Netw Open.* 2024;7(7):e2421010. doi:10.1001/jamanetworkopen.2024.21010
10. Morenz AM, Liao JM, Au DH, Hayes SA. Area-Level Socioeconomic Disadvantage and Health Care Spending: A Systematic Review. *JAMA Netw Open.* 2024;7(2):e2356121. doi:10.1001/jamanetworkopen.2023.56121

© 2025 Nananukul N et al. *JAMA Network Open*.

11. https://sample-size.net/logistic-regression-effect-size/
12. https://geopandas.org/en/stable/

13. https://matplotlib.org/

14. https://seaborn.pydata.org/

© 2025 Nananukul N et al. *JAMA Network Open*.
