# Supplementary material for: Medical School Ranking and Neighborhood Characteristics of Initial Practice Location Among Physicians
Source: JAMA Netw Open. 2025 May 28;8(5):e2512474. doi: 10.1001/jamanetworkopen.2025.12474 (PMC12120638; doi:10.1001/jamanetworkopen.2025.12474)
Supplement: Supplement 2. — Data Sharing Statement [file jamanetwopen-e2512474-s002.pdf]

Data Sharing Statement

Nananukul. Medical School Ranking and Neighborhood Characteristics of Initial Practice Location Among Physicians. *JAMA Netw Open*. Published May 28, 2025. doi:10.1001/jamanetworkopen.2025.12474

Data
Data available: Yes
Data types: Data (not involving human participants)
How to access data: Please send request for data to kejriwal@isi.edu
When available: With publication

Supporting Documents
Document types: Statistical/analytic code
How to access documents: Please send request for code to kejriwal@isi.edu. We will also make it available in a github repository with publication.
When available: With publication

Additional Information
Who can access the data: Everyone requesting the data
Types of analyses: Any purpose
Mechanisms of data availability: Without investigator support
